# Bioactivity of *Falkenbergia rufolanosa* Methanolic Extract: Assessment of Its Effect on Methyl-Thiophanate Induced Bone and Blood Disorders

**DOI:** 10.3390/ph16040529

**Published:** 2023-04-01

**Authors:** Amal Feki, Intissar Kammoun, Malek Eleroui, Rim Kallel, Fatma Megdiche, Liwa Hariz, Tahia Boudawara, Choumous Kallel, Hatem Kallel, Jean Marc Pujo, Ibtissem Ben Amara

**Affiliations:** 1 Laboratory of Medicinal and Environment Chemistry, University of Sfax, Higher Institute of Biotechnology, Sfax 3000, Tunisia; 2 Laboratory of Anatomopathology, CHU Habib Bourguiba, University of Sfax, Sfax 3029, Tunisia; 3 Laboratory of Hematology, CHU Habib Bourguiba, University of Sfax, Sfax 3029, Tunisia; 4 Laboratory of Anatomy, Faculty of Medicine of Sfax, Department of Orthopedic and Traumatological Surgery, CHU Habib Bourguiba, Sfax 3029, Tunisia; 5 Intensive Care Unit, Cayenne General Hospital, Cayenne 97300, French Guiana; 6 Tropical Biome and Immunopathology CNRS UMR-9017, Inserm U 1019, University of Guyane, Cayenne 97300, French Guiana; 7 Emergency Department, Cayenne General Hospital, Cayenne 97300, French Guiana

**Keywords:** *Falkenbergia rufolanosa*, Methyl-thiophanate, oxidative stress, genotoxicity, DNA degradation, mineral composition

## Abstract

This study aimed to evaluate the potentiality of a mineral and antioxidant-rich methanolic extract of the red marine alga *Falkenbergia rufolanosa* (FRE) against methyl-thiophanate (MT)-induced toxicity in adult rats. The animals were allocated into four groups: controls, MT (300 mg/kg), MT + FRE, and FRE-treated group for 7 days. Our results demonstrated severe mineral perturbations due to MT treatment, especially in calcium and phosphorus levels in plasma, urine, and bone. Similarly, the hematological analysis revealed increased red blood cells, platelets, and white blood cells associated with striking genotoxicity. Interestingly, a significant rise in lipid peroxidation and advanced oxidation protein products level in erythrocytes and bone were noted. Meanwhile, a depletion of the antioxidant status in both tissues occurred. These biochemical alterations were in harmony with DNA degradation and histological variation in bone and blood. In the other trend, data showed that treatment with alga improved MT-induced hematotoxicity, genotoxicity, and oxidative stress in the blood and bone. Osteo-mineral metabolism and bone histo-architecture were also noted. In conclusion, these findings demonstrated that the red alga *Falkenbergia rufolanosa* is a potent source of antioxidant and antibacterial agents, as revealed by the in vitro analysis.

## 1. Introduction

Excessive pesticide use resulted in several diseases’ prevalence [1]. It is established that many pesticides can produce massive amounts of free radicals, which could damage exogenous cell components, impairing antioxidant status [2].

Methyl thiophanate (MT), a thioallophanate compound, is a fungicide widely used to control acute fungal diseases of crops and weeds [3]. In humans and other mammals, MT is metabolized to benzimidazole compounds, including methyl-2-benzimidazole carbamate (carbendazim) [4]. This agrochemical compound may induce DNA damage in human lymphocytes [5], degradation of seminiferous epithelium, and inhibition of the expression of steroid receptors, inducing infertility [4]. Bolognesi [6] revealed that this fungicide might cause higher genotoxicity than those predicted from conventional toxicity tests for other pesticides. According to Ben Amara et al. [4], MT-induced toxicity seems to generate large amounts of reactive oxygen species (ROS), leading to severe genotoxicity.

Previous studies indicated that antioxidant components such as phenolics, flavonoids, anthocyanins, and minerals could help treat and prevent oxidative stress and ROS-related diseases [7]. Recently, seaweeds have attracted interest for their rich composition in several minerals, proteins, vitamins, fibers, trace elements, and bioactive compounds [8]. The most common minerals in seafood are magnesium, sodium, calcium, phosphorus, iron, potassium, and copper. Such variety could develop new drugs and healthy food [9]. *Falkenbergia rufolanosa* (*F. rufolanosa*) is a Rhodophyta of the Bonnemaisoniales order, spread along all Mediterranean coasts and the Atlantic islands of Madeira and the Canaries [10].

To the best of our knowledge, no investigation has so far reported on the protective effects of *F. rufolanosa* against blood, bone mineral composition, and oxidative disturbances induced by MT in adult female rats.

## 2. Results

### 2.1. In Vitro Study

#### 2.1.1. Yield and Chemical Composition

The yield and pH of FRE were about 2.2% and 7.67, respectively. Moisture, proteins, and ash contents of FRE were about 2.7%, 43.95%, and 69.9%, respectively. Polysaccharides fraction represents 52.25% of alga extract.

#### 2.1.2. Amounts of Polyphenols, Flavonoids, Anthocyanins, and Vitamin C

The total phenolic compounds were estimated as equivalent to 14.2 mg of gallic acid/g in the FRE. The total flavonoid content was approximately equivalent to 5.95 mg of quercetin/g of extract. The total anthocyanin content was 0.868 mg/100 g of dry extract. Vitamin C content represents about 650 mg/kg of extract.

#### 2.1.3. Mineral Contents

Our results showed that the red alga was rich in mineral compounds. Our findings indicated that FRE has high amounts of Mg (677.7 ± 0.07 mg/L), Na (282.11 ± 4.03 mg/L), K (2409.41 ± 3.17 mg/L), and contained small amounts of Zn (0.91 ± 0.91 mg/L) and Fe (22.67 ± 2.27 mg/L).

#### 2.1.4. The Antioxidant Activity

**Figure 1:**
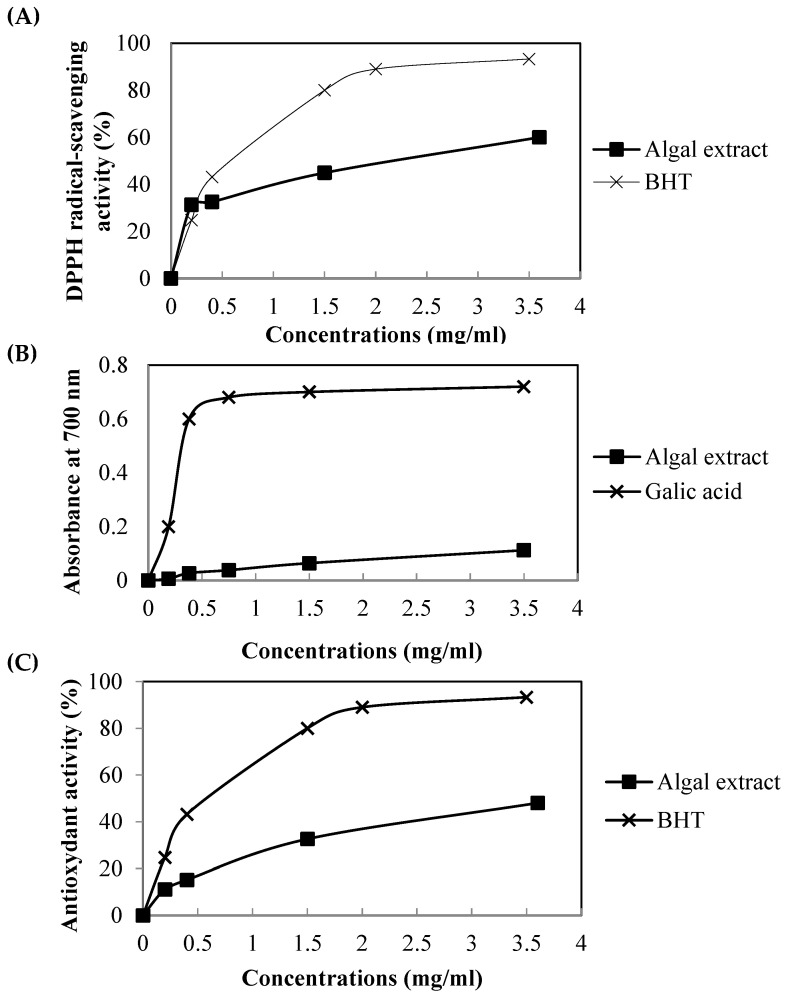
(**A**) DPPH radical-scavenging activity, (**B**) Reducing power; (**C**) ß-carotene bleaching activity of FRE at different concentrations. Vitamin C, gallic acid and BHT were used as positive controls. All analysis was carried out in triplicate.

The scavenging capacity of FRE based on DPPH inhibition is shown in Figure 1A. The results indicated that alga at high concentration (3.5 mg/mL) exhibited a substantial antiradical activity with about 60%, but still lower than that of the antioxidant standards, BHT (90%).

The reducing power of FRE is shown in Figure 1B. The result suggested that the reducing power values of alga increased with increasing concentration. However, gallic acid, used as a standard reference, showed higher reducing power than the red seaweed extracts.

The antioxidant activity through the β-carotene/linoleate model system of alga extract was assayed and compared to BHT (Figure 1C). FRE radical scavenging activity steadily increased in a dose-dependent manner (0, 0.2, 0.4, 1.5, and 3.5 mg/mL) with 0%, 12%, 15%, 33%, and 50%, respectively, but lower than that of BHT.

#### 2.1.5. Antibacterial Activity of FRE

The antibacterial activity of FRE is checked against six strains of bacteria, as presented in Table 1. Alga showed significant bactericidal effects at a concentration of 10 mg/mL with inhibitory zones ranging from 6.5 to 11 mm.

### 2.2. In Vivo Study

#### 2.2.1. Effects on the General Health

During the experimental period, MT-treated rats showed no mortality but varying degrees of clinical signs, including vomiting, reduced activity, conjunctivitis, and diarrhea. However, higher vital activity in control and co-treated rats was noted compared to MT-treated rats.

#### 2.2.2. Food and Water Intake

Food and water consumption of MT-treated rats were reduced by 24 and 23%, respectively. On the other hand, co-treatment with FRE improved these results, which reached near-normal values. The administration of FRE only in the diet did not affect the daily food and water intake (Table 2).

The body weight of rats was monitored daily. At the beginning of the treatment (day 0), the body weight of different experimental groups are approximatively near (Table 2). At the end of treatment, rats exposed to MT acquired a decrease in their body weight when compared to the controls. Conversely, co-administration of FRE partially increased their body weight compared to MT-treated rats. On the other hand, rats treated only with FRE showed no significant differences compared to the control group (Table 2). Similarly, a considerable decrease in femur weight was noted in the MT-treated group compared to the controls. The addition of FRE improved femur weight.

#### 2.2.3. Determination of Hematological Parameters

Compared with the control group, a decrease in RBC, Hb, and platelet contents was noted, accompanied by an increase in WBC counts, in rats exposed to MT (Table 3). No changes in MCV, MCH, and MCHC in MT-treated rats were observed. The changes induced by the administration of MT were significantly reversed by co-treatment with algal extract compared to the controls.

#### 2.2.4. Enmeration of WBC Formula

**Figure 2:**
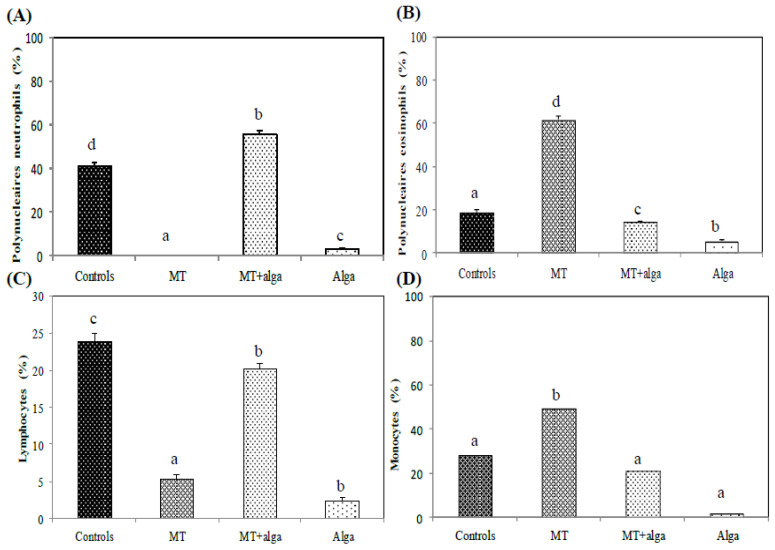
Numeration of White Blood Cells formula in different experimental groups of rats. (**A**) Polynucleaires neutrophils; (**B**) Polynucleaires eosinophils; (**C**) Lymphocytes; (**D**) Monocytes. ^a,b,c^ indicate significant differences between experimental groups (*p* < 0.05).

The result revealed an increase in WBC in rats exposed to MT, as noted in Figure 2. The enumeration of the WBC formula demonstrated an increase in eosinophils and monocytes and a decrease in the number of neutrophils and lymphocytes in the MT-treated rats (Figure 2). Contrarily, co-treatment with FRE induced an amelioration of these parameters compared to the MT group.

#### 2.2.5. Achievement of Blood Smears

**Figure 3:**
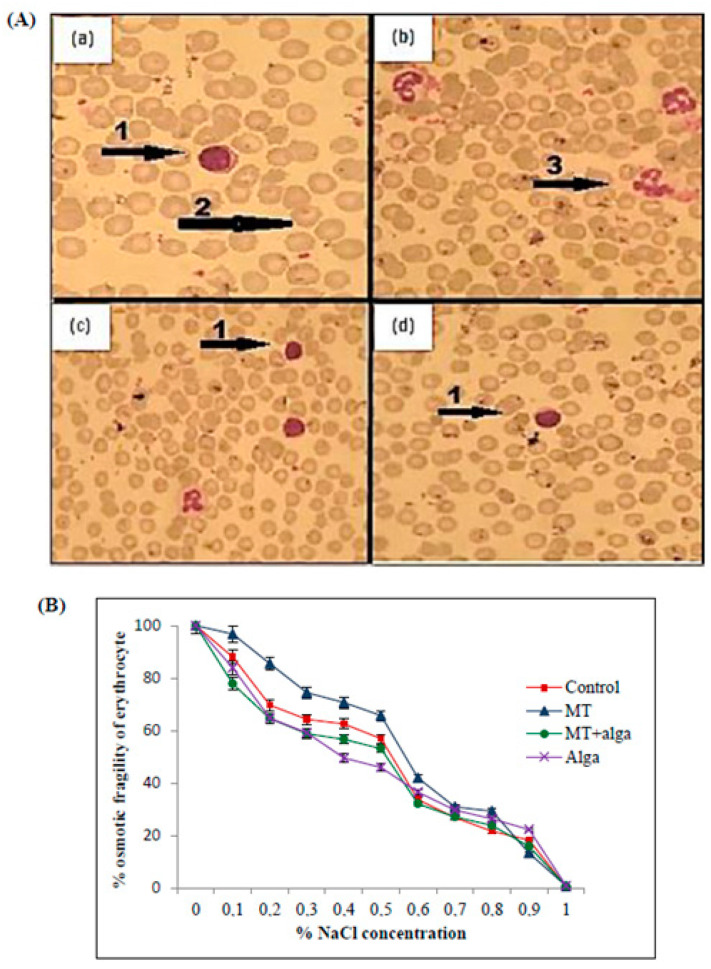
(**A**) Blood smear of adult rats (×100) in different experimental groups. (**a**) Control group; (**b**) rats treated with MT; (**c**) rats treated with MT + FRE; (**d**) rats treated with FRE. 1: Mononuclear leukocyte; 2: erythrocyte; 3: apoptotic cell. (**B**) Evolution of erythrocyte osmotic fragility in adult rats, controlled and treated for 7 days with MT, MT + FRE, and FRE.

In the MT-treated rats, blood smears showed the presence of some necrotic and apoptotic cells (Figure 3A). However, co-treatment with FRE improved these parameters compared to the MT group.

As shown in Figure 3B, the group treated with MT showed an increase in osmotic fragility compared to untreated rats. In contrast, the co-treatment of MT-treated rats with FRE ameliorates this percentage.

#### 2.2.6. MN Assay

**Figure 4:**
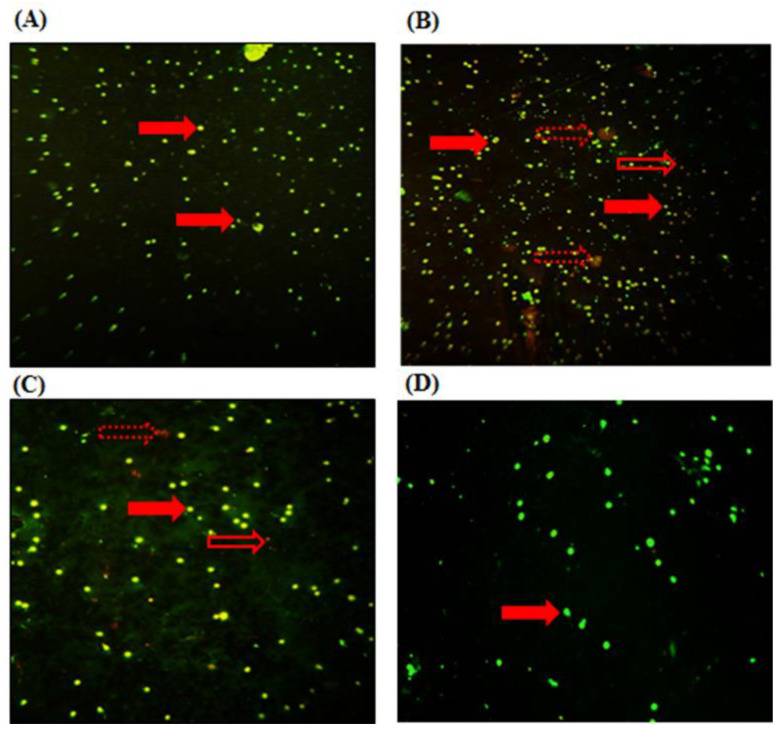
Micronucleus test of controls and treated rats for 7 days with MT, and MT co-administrated with alga. (**A**) Control group; (**B**) rats treated with MT; (**C**) rats treated with MT + FRE; (**D**) rats treated with FRE. Arrows indicate the following: 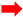 nucleus of WBC; 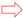 necrotic cells; 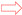 apoptotic cells.

Compared to the control group, MT-treated rats induced an increase in WBC (green/yellow fluorescence (intact DNA) with the presence of a few nuclear fragmentations in WBC revealed by red/orange fluorescence (damaged DNA) (Figure 4B). However, algal supplementation improved mean cells’ damage and decreased the frequency of damaged DNA (Figure 4C). No significant difference in WBC morphology was noted in rats treated with the alga extract alone (Figure 4D).

#### 2.2.7. Biochemical Assays

##### Mineral Contents Levels in Plasma, Urine, and Femurs

As shown in Table 4, MT induced a significant perturbation in the plasma, bone, and urine mineral contents compared to the controls. After the co-administration of FRE, mineral contents were regulated, especially for calcium and phosphorus, compared to the MT group.

##### Determination of Oxidative Stress Markers

Our results revealed an increase in MDA and AOPP levels in erythrocytes and bone and a decrease in GSH levels in both tissues in the MT-treated group compared to the controls (Table 5). Conversely, co-treatment with alga extract significantly modulated these parameters compared to the MT-treated group. No significant difference was noted in erythrocytes and bone in the alga group.

In addition, a significant decline in the activities of CAT, SOD, and GPx in erythrocytes and bone of adult rats treated with MT was noted (Table 5). The administration of FRE significantly ameliorated the activities of these enzymes.

##### Determination of Lactate Dehydrogenase Activity

**Figure 5:**
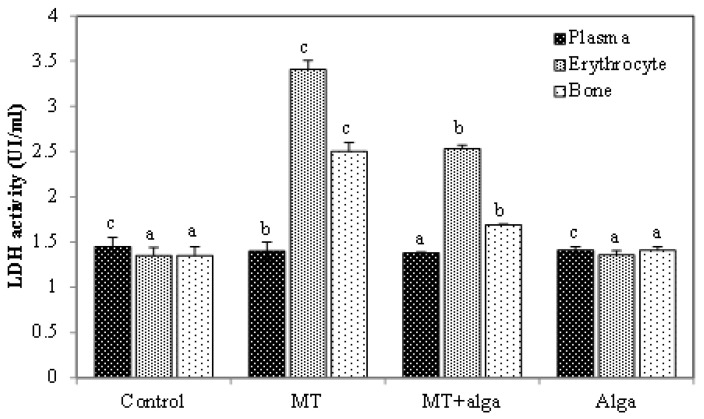
Lactate dehydrogenase activity in plasma, erythrocytes and bone in different groups of rats. ^a,b,c^ indicate significant differences between experimental groups (*p* < 0.05).

LDH activity was increased in erythrocytes and bone and decreased in plasma in MT-treated rats (Figure 5). Co-treatment with FRE restored LDH activity in both tissues.

#### 2.2.8. Histological Studies

**Figure 6:**
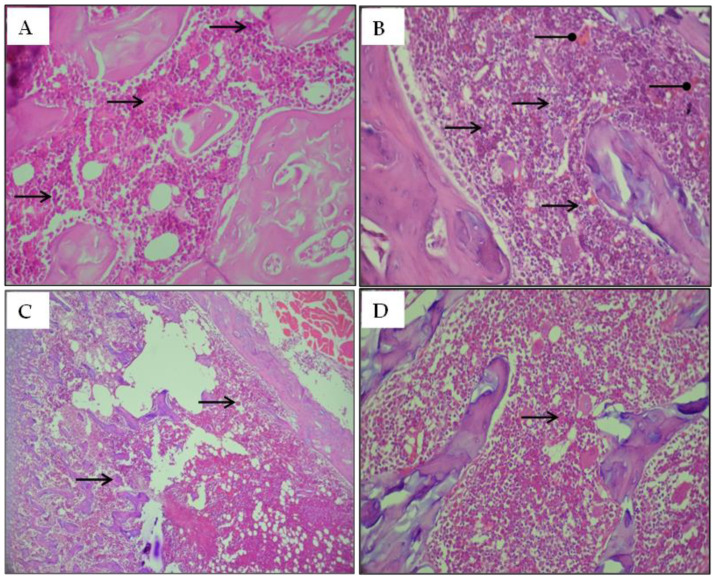
Optical microscopy histological sections of the bone marrow stained with hematoxylin-eosin (H&E) of adult rats. (**A**) Controls group; (**B**) Rats treated with MT; (**C**) Rats treated with MT + FRE and (**D**) Rats treated with FRE; Arrows indicate the following: 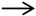: Megakaryocytic cell; 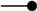: Apoptosis.

Bone marrow histological sections in MT-treated rats indicated an elevation in the number of megakaryocytic cells and numerous apoptotic cells (Figure 6B). Conversely, co-treatment with alga alleviated such modifications in bone marrow tissue similar to that in control rats (Figure 6C) and in rats treated only with the algal extract (Figure 6D).

**Figure 7:**
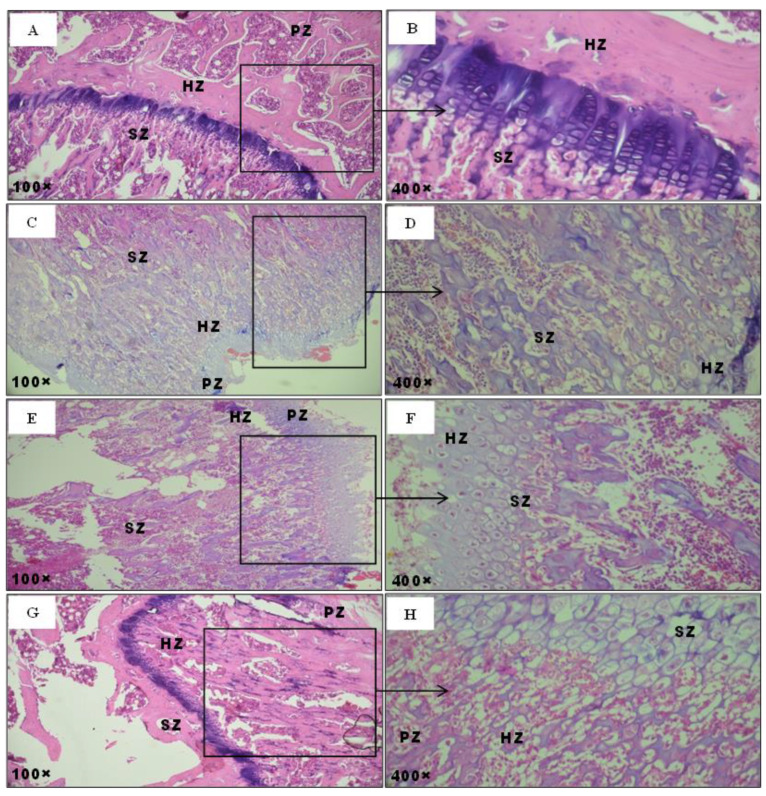
Optical microscopy histological sections of the femur stained with hematoxylin-eosin (H&E) of adult rats. (**A**,**B**) controls group; (**C**,**D**) rats treated with MT; (**E**,**F**) rats treated with MT + FRE; (**G**,**H**) rats treated with FRE. Arrows indicate the following: PZ: proliferative zone; HZ: hypertrophic zone; SZ: spongy zone.

Femur histological sections indicated a normal histo-architecture in the bone in control rats (Figure 7). The hypertrophic zone (HZ), spongy zone (SZ), and proliferative zone (PZ), the three classical zones in femur metaphysis, contained flattened chondrocytes parallel to the growth axis. Bone trabeculae in the primary spongiosum of control rats were organized in parallel to the columns of proliferating chondrocytes (Figure 7A,B). Conversely, in the femur sections of MT-treated rats, bone trabeculae were fewer and more fragmented than those of the controls (Figure 7C,D). The growth plate in the treated rats, which could be explained by the abundance of the cartilaginous matrix and lack of chondrocytes, was highly disorganized and failed to form columns. Also, hypertrophic chondrocyte differentiation was developed compared to the control rats (Figure 7C,D). On the other hand, co-treated rats with the algal extract demonstrated a slight amelioration of the alterations in the MT-treated group (Figure 7E,F).

**Figure 8:**
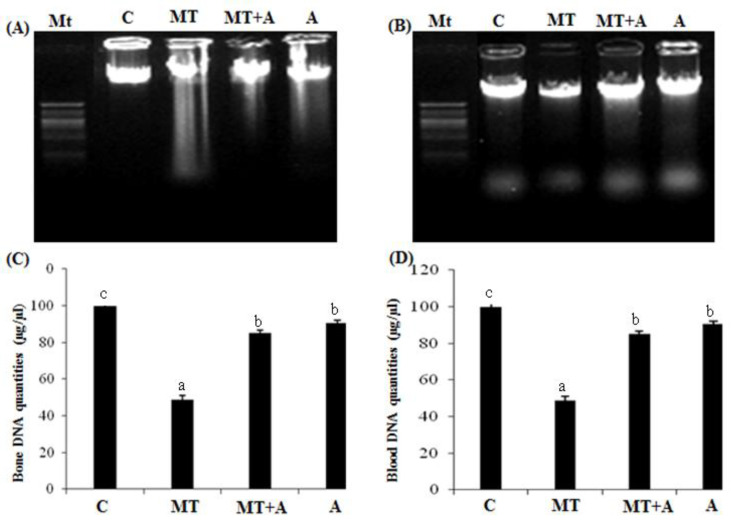
(**A**) Agarose gel electrophoresis of the bone DNA fragmentation. MT, marker (1 kb DNA ladder); lane 1, controls group; lane 2, MT treated group (MT); lane 3, MT + FRE-treated group (MT + A); lane 4, FRE-treated group; (**B**) Agarose gel electrophoresis of the WBCs DNA fragmentation. MT, marker (1 kb DNA ladder); lane 1, control group (C); lane 2, MT treated group (MT); lane 3, MT + FRE-treated group (MT + A); lane 4, FRE-treated group (A); (**C**) Bone DNA quantities of controls (C) and rats treated with MT (MT), MT + FRE (MT + A), and FRE (A) after 7 days; (**D**) Bloob DNA quantitie controls s of (C) and rats treated with MT (MT), MT + FRE (MT + A), and FRE (A) after 7 days. ^a,b,c^ indicate significant differences between experimental groups (*p* < 0.05).

Finally, no significant differences in femur sections of rats treated only with the algal extract were noted compared to the controls (Figure 8A,C). However, co-administration with FRE induced a significant depletion in the amount of genomic DNA breakdown when compared to the MT-treated group (Figure 8A,C). No significant difference was noted in rats treated only with alga extract compared to the control group (Figure 8A,C).

## 3. Discussion

Nowadays, pesticide exposure presents a severe health threat. In this context, methyl-thiophanate (MT), one of the main fungicides used worldwide, is classified as a category-III acute inhalation toxicant. Referring to the literature, the derived elements from these agrochemicals are principally retained in the bone and blood tissues. Our study suggests that rats’ exposure to MT for seven days induced a decrease in their body and femur weights when compared to the controls. This perturbation could be attributed to the reduction in food consumption, as demonstrated by our results. Co-administration with FRE significantly improved these parameters. We hypothesized that the richness of red alga in mineral compounds (Ca, P, Na, Mg, Fe, K, and Zn) might compensate for the loss of body weight engendered by MT exposure. According to Jaballi et al. [7], a mineral-rich extract derived from a red marine alga, *Chondrus canaliculatus*, could be used as a dietary supplement to prevent bone mineral loss and stimulate body growth. Interestingly, seaweeds are also enriched with other components, such as dietary fiber and resistant protein, that may flow through the intestine without being absorbed [11].

Bone is a composite natural material consisting of collagen fibers and hydroxyapatite crystals containing inorganic components, mainly calcium and phosphorus. Conventionally, calcium and phosphorus are considered phenotype markers of bone formation [12]. In our experimental study, the exposure of rats to MT affected the bone mineral composition owing to a significant decline in calcium and phosphorus contents. Paradoxically, these two mineral compounds increased in plasma and urine compared to the controls. These results were in harmony with previous studies by Andrews et al. [13], who reported a disturbance in calcium and phosphorus of rats exposed to dimethoate.

The main link between bone and erythrocytes is because bone marrow is the site of hematopoiesis, the process by which the cellular components of blood are generated [14]. In a biological context, the mature mammalian erythrocyte lacks a nucleus and mitochondria. In such conditions, RBCs cannot use oxygen for themselves; thus, all the oxygen absorbed by the hemoglobin of RBC is transported to the tissues [15]. In this trend, the hematological results revealed a significant decrease in the number of erythrocytes and in the Hb level. There was a reduction in the number of platelets, probably induced by oxidative stress associated with MT. In addition, our experiment showed a significant increase in the total number of leukocytes. This hematological perturbation could be the result of the excessive production of blood cellular components by the bone marrow, as reported by Elwej et al. [16]. In addition, the numeration of the WBCs formula noted an increase in the number of eosinophils and monocyte cells, mainly correlated to inflammation [17]. Meanwhile, depletion in the numbers of neutrophils and lymphocytes was seen. The drop in the metabolic rate of neutrophils and lymphocytes confirmed the presence of necrotic and apoptotic cells and a DNA breakdown within the MN assay, suggesting, thereby, the pro-oxidant and genotoxic effects of MT [18,19]. The impairment of hematological parameters might be due to oxidative stress probably generated by this fungicide. Erythrocytes are known for their sensitivity to oxidative injury [20]. Their membranes are susceptible to covalent damage, including cross-linking and aggregation by free radical overproduction resulting from Hb auto-oxidation. We can conclude that Hb in erythrocytes is a major source of radical production. Agreeing with all mentioned data, Shiozawa et al. [21] reported that changes in bone mass associated with a moderate increase in Ht could be theoretically related to bone morrow injuries through ROS generated by oxidative stress.

ROS are oxygen-containing molecules in various biological processes, such as normal cellular signaling and immune defense. As mentioned by Vener et al. [22], elevated levels of ROS could be the crucial reason for the development of cancer, solid tumors, and hematological malignancies [23]. Accordingly, an imbalance between oxidants and cell antioxidants due to oxidative stress installation lead to ROS accumulation leading to biomolecular oxidation, including lipid peroxidation, protein oxidation, and DNA breakdown [24]. In the present study, exposure to MT revealed an increase in MDA, AOPP, and LDH activities in erythrocytes and bone, which could be evidenced by lipid alteration and protein oxidation involved by free radicals. Generally, the increase in LDH activity, osmotic fragility, and MDA levels indicated intravascular hemolysis [25]. Thus, erythrocytes are extremely sensitive to oxidative injury because their massive rate of oxidative metabolic activity gives them the voidability to cope with oxidative stress and evenly compromise cell viability [26,27]. The antioxidant status in both tissues was also evaluated to substantiate the mechanisms underlying MT toxicity. Our results indicated a decrease in SOD and CAT activities in erythrocytes and bone. This reduction of CAT activity might reflect the inability of erythrocytes to eliminate H_2_O_2_ produced by MT. Similarly, our results indicated a decrease in GPx and GSH levels in both tissues. The exhaustion of GPx activity and GSH level in the bone and erythrocyte could be marked by the disruption in the production cycle of GSH caused by the over-accumulation of free radicals generated by MT [7]. In fact, as demonstrate by our results, the generation of ROS induced by MT contributed to hematotoxicity and genotoxicity. Accordingly, MT treatment resulted in blood DNA fragmentation with a subsequent formation of a DNA smear on agarose gel, a hallmark feature of necrosis in blood. These data were substantiated by blood smears histopathological observations which were characterized by necrotic cells, confirming DNA fragmentation test.

Our data was proved by the MT’s histological sections, which showed a deep disturbance in the different femur zones and a depletion of the growth plate in the cartilaginous matrix, a sign of decalcification. Accordingly, disorders in bone formation could be explained by an accumulation of ROS in the bone marrow reflecting changes in mineral composition [12,13,14,15,16,17,18,19,20,21,22,23,24,25,26,27,28].

Although the detailed mechanism of MT-mediated toxicity is still unknown, the present study showed that this fungicide-induced oxidative stress produces deleterious modifications in membranes, proteins, enzymes, and DNA. Our data demonstrated that co-administration of FRE significantly improved the mentioned perturbations in blood and bone. In fact, FRE was effective in alleviating hematotoxicity induced by MT, as reflected by the improvement in hematological parameters (erythrocyte, Ht, Hb, and osmotic fragility). This might be due to flavonoids present in alga extract that helped to prevent membrane fragility. The leukocytosis effect was also reduced by FRE administration, as evidenced by a decrease in WBC counts, confirming the anti-inflammatory property of this alga. In this attempt, the abundance of mineral compounds in seaweeds might exhibit a significant role in restraining human tissues and regulating vital reactions [10]. These findings are in harmony with the high ash content in FRE, indicating the presence of appreciable amounts of various minerals reacting as critical components of cellular antioxidant enzymes [11]. Interestingly, the administration of FRE had a potent protective effect against oxidative stress in both tissues induced by MT, as revealed by a remarkable decrease in MDA and AOPP and an increase in antioxidant enzyme activities and GSH. The regulation of the altered antioxidant status and peroxidative damage in erythrocytes and bone could be related to its antioxidant and antiperoxidative properties and its potential role in the defense against free radicals. In fact, our in vitro study affirmed that FRE is rich in polyphenols and flavonoids. These compounds have been reported to exert several medicinal properties and biological activities, including antioxidant, free radical scavenging ability, anti-inflammatory, and anti-carcinogenic effects. Flavonoids might act as hydrogen donors to free radicals, thereby blocking ROS chain reactions and suppressing lipid peroxidation [29,30]. Meanwhile, polyphenols significantly enhanced the ability of RBCs to form a barrier against ROS and improved their antioxidant activity [31]. Previous studies have shown that these compounds (flavonoids and polyphenols) inhibit free radical formation and their propagation reactions, thereby allowing the recovery from cell injuries and DNA protection before ROS can cause any damage [32]. Lastly, vitamin C, the powerful primary antioxidant compound, reinforced the potentiality of FRE to inhibit ROS generation and protect the body against free radicals and diseases and reduce the risk of inflammation [33,34].

## 4. Materials and Methods

### 4.1. Material

Fresh fronds of *F. rufolanosa* were collected from Sfax City (southern Tunisia) in February 2018. After collection, the algal material was washed with tap water many times and washed twice with distilled water to remove epiphytes, salts, and sand. Next, they were air-dried in the shade and ground by a blender to give small pieces (2 mm) and then stored in plastic bags at room temperature in a dry, dark place before use.

### 4.2. Extraction of F. rufolanosa

The air-dried frond powder (30 g) was extracted by maceration. As the first step, we put 30 g of crude extracts of dried alga powder in 200 mL of methanol solvent. Secondly, the mixture was incubated for 24 h with stirring at room temperature. After filtration with Whatman filter paper, the solution was evaporated using a Rotary evaporator. As the final step, each dried residue was re-dissolved in the corresponding solvent and then stored at 4 °C.

### 4.3. Physicochemical Properties of FRE

The moisture and ash contents were determined according to the AOAC standard methods [35]. Total carbohydrates were determined by the phenol-sulphuric acid method [36]. Protein content was estimated by the method of Bradford [37]. The absorbance was measured at 595 nm. The pH (1% solution at 25 °C) was measured using a digital pH meter (Systronics Instruments, Karnataka-India) with completely immersing the glass electrode into the solution.

According to Aliou and Lamine [38] method, iron (Fe), magnesium (Mg), zinc (Zn), potassium (K), and sodium (Na) were measured after nitroperchloric mineralization (2/1 V) of the algal extract by atomic absorption spectroscopy (model Thermo-Scientific ICE 3000, Sherwood Scientific Ltd., Cambridge, UK). The wavelengths of the elements to be analyzed were as follows: Fe (248.3 nm), Mg (285.2 nm), Zn (213.9 nm), K (766.5 nm), and Na (589 nm). Calcium (Ca) and phosphorus (P) were measured using commercial reagent kits (Biocon, refs. 2004 and 1904, respectively).

### 4.4. Determination of Total Phenolic Content (TPC)

Total phenolics were determined using the Folin–Ciocalteu reagent as described by Velioglu et al. [39] with slight modifications. After incubation for 90 min in the dark, the absorbance was measured at 725 nm using Jenway 6300 spectrophotometer (Bibby scientific limited, ST15 OSA, UK). Total phenolic contents were expressed as mg of gallic acid/g of dry alga.

### 4.5. Determination of Total Flavonoid Content

Flavonoid content in the alga extract was determined using the Aluminum chloride (AlCl_3_) colorimetric method according to Arvouet–Grand et al. [40]. The absorbance was determined at 415 nm using the Jenway 6300 spectrophotometer after 15 min incubation at room temperature. Quercetin was considered as a standard, and the results were expressed as mg of quercetin/g of alga extract.

### 4.6. Determination of Total Anthocyanin Content (TAC)

The total anthocyanin content was determined by the monomeric anthocyanin method of Wrolstad et al. [41]. The absorbance was measured simultaneously at 510 and 700 nm. Total anthocyanin content was expressed as mg of cyanidin-3-glucoside equivalents per 100 g extract.

### 4.7. Determination of Total Vitamin C Content

Ascorbic acid determination was performed as described by Jacques–Silva et al. [42]. The reaction product was determined using a color reagent containing dinitrophenylhydrazine and CuSO_4_. The data were expressed as mg ascorbic acid/kg of dry extract.

### 4.8. Determination of Antioxidative Activities In Vitro

#### 4.8.1. DPPH Radical-Scavenging Assay

DPPH radical-scavenging activity of alga was determined, as described by Bersuder et al. [43]. Briefly, samples of alga extract and BHT used as standards were established at different concentrations (0.2–3.5 mg/mL). The reduction of DPPH radical was measured at 517 nm.

#### 4.8.2. Reducing Power Assay

The ability of alga to reduce iron (III) was determined according to the method of Oyaizu [44]. The absorbance was measured at 700 nm. The control was made in the same manner, except that distilled water was used instead of the sample. Gallic acid was used as an antioxidant reference.

#### 4.8.3. β-Carotene Bleaching (BCB) Assay

The ability of the extract to prevent the bleaching of β-carotene was determined by the method of Koleva et al. [45]. The absorbance was measured at 470 nm before and after incubation of the sample with β-carotene/linoleic acid emulsion. BHA was used as a positive standard.

### 4.9. Antimicrobial Activity

Antimicrobial activities of alga methanolic extract were tested against four strains of Gram-positive bacteria: *Micrococcus luteus*, *Enterococcus Faecalis*, *Actinomyces* sp., and *Listeria Monocytogenes* and 2 strains of Gram-negative bacteria *Enterobacter*, *klebsiella Pneumonia*, and *Salmonella Typhimurium*.

The agar diffusion method was followed for the antibacterial and antifungal susceptibility test [46]. Petri plates were prepared by pouring 10 mL of Mueller Hinton Agar for bacteria and allowed to solidify. Fronds were dried, and 0.1 mL of inoculum suspension of bacteria was poured and uniformly spread. After drying the rest, an equal amount of extract was poured into the holes. Then, the Petri plates were applied, and the plates were incubated at 37 °C for 24 h. The inhibition zone was measured from the edge of the disc to the inner margin of the surrounding pathogens.

### 4.10. Animals and Experimental Design

Wistar adult rats weighing about 170 ± 10 g, obtained from the Central Pharmacy (SIPHAT, Tunisia), were housed in clean polyethylene cages with an acclimate-controlled facility and a constant light-dark cycle at a temperature of 22 °C ± 2 and humidity of 40%. LD50 of MT was evaluated (1000 mg/kg) in our laboratory by Ben Amara et al. [4]. Rats were randomly divided into four groups of six animals each: rats of group 1 (control group) received oil corn injection, used as a vehicle; group 2 received by intraperitoneal a single injection of 300 mg/kg of MT; group 3 received daily both MT (300 mg/kg) by intraperitoneal injection and alga methanolic extract (100 mg/kg of the algal extract) via the alimentation; group 4 received only alga (150 mg/kg of the algal extract) added to their diet.

The treatments were carried out for seven days. The treatment period of MT was selected on the basis of previous studies to be toxic but not lethal [4]. The dose of alga methanolic extract was shown in previous studies to induce benefic effects without being toxic [7].

The experimental procedures were carried out according to the Natural Health Institute of Health Guidelines for Animal Care and approved by the local Ethical Committee. All animal procedures were conducted in strict conformity with the “Institute’s ethical committee guidelines” for the Care and Use of laboratory animals.

During the treatment period, food (g/day/rat), water intake (ml/day/rat), and body weight (g) of the animals were monitored daily.

#### 4.10.1. Blood and Organ Preparation

At the end of the experiment, animals were sacrificed by cervical decapitation to avoid stress. Some blood samples were collected for the determination of hematological parameters and micronucleus (MN) tests. After centrifugation, the sediment-containing erythrocytes were suspended in phosphate-buffered saline solution (0.9% NaCl in 0.01 M phosphate buffer, pH 7.4) and centrifuged for erythrocytes extraction as described by Sinha et al. [47].

Femurs were dissected out, and the surrounding muscles and connective tissues were removed. Then, the bone marrow of the remaining ones was removed according to the method described by Amend et al. [48] and Jaballi et al. [7]. After that, the white femur was taken (without bone marrow) and homogenized with 2 mL of 0.1 M Tris–HCl buffer (pH 7.2) using a mortar and pestle, according to Ramajayam et al. [28]. The homogenates were centrifuged at 10,000× *g* for 15 min at 4 °C, and the resulting supernatants were used for the biochemical assays. All samples were weighed. Some of them were immediately fixed in 10% formalin solution for histological examination, and others were mineralized to serve for mineral determination.

#### 4.10.2. Biochemical Assays

##### Determination of Hematological Parameters

White blood cells (WBC), red blood cells (RBC), platelets (Plt), hematocrit (Ht), hemoglobin (Hb), mean corpuscular volume (MCV), mean corpuscular hemoglobin (MCH) and mean corpuscular hemoglobin concentration (MCHC) were analyzed by an electronic automate Coulter MAXM (Beckman Coulter, Inc., Fullerton, CA, USA).

##### Osmotic Fragility Test

The osmotic fragility of erythrocytes was measured using NaCl solutions at different concentrations (0.1–0.9% of NaCl). The Hb content of the supernatant was determined spectrophotometrically at 540 nm. The amount of lysis in each tube was compared with a 0.1% NaCl tube corresponding to 100% lysis.

##### Achievement of Blood Smears

A drop of fresh blood was at first spread on a slide, and then May Grunwald was added for 2 min and rinsed with water. After that, Giemsa was added for staining. Different blood cells were visualized using an optical microscope at magnification (×100), and the WBC formula was counted.

##### MN Assay in Peripheral Blood

Peripheral WBC fractions were extracted at the middle of the Ficoll gradient. The morphology of the cell’s nuclei was observed using a fluorescence microscope at an excitation wavelength of 520–560 nm. For that, slides were incubated with 500 μL of acridine orange (0.5 μg/mL) for 10 min at room temperature, then they were dried at 37 °C, and finally, they were washed with PBS buffer.

##### Mineral Levels in the Bone, Blood, and Urine

Magnesium, potassium, sodium, calcium, and phosphorus levels in the femur, plasma, and urine were measured as described previously for algal extract (see the “Mineral Content Assay” section).

##### Determination of Malondialdehyde and Advanced Oxidation Protein Products Levels

The malondialdehyde (MDA) levels, and index of lipid peroxidation, were estimated spectrophotometrically referring to Draper and Hadley [49] methods. The MDA values were calculated using 1,1,3,3-tetra ethoxy propane (TEP) used as standard. MDA levels were expressed as nmoles of MDA/mg tissue.

Advanced oxidation protein products (AOPP) levels were measured according to Witko [50] method. The absorbance was determined at 340 nm. The concentration of AOPP for each sample was calculated using the extinction coefficient of 261 cm^−1^ mM^−1^. AOPP levels were expressed as μmoles/mg protein.

##### Determination of Enzymatic and Non-Enzymatic Antioxidants

Catalase (CAT) activity was determined by the decomposition of hydrogen peroxide according to the method of Aebi [51]. Changes in absorbance due to H_2_O_2_ degradation were determined spectrophotometrically at 240 nm. The limit of detection of the method is 0.98 mmoles. The enzyme activity was expressed as μmol H_2_O_2_ consumed/min/mg of protein.

Superoxide dismutase (SOD) activity was estimated according to the method of Beauchamp and Fridovich [52]. Units of SOD activity were expressed as the amount of enzyme required to inhibit the reduction of nitro blue tetrazolium (NBT) by 50%. The developed blue color in the reaction was measured at 560 nm, and the activity was expressed as units/mg of protein.

Reduced glutathione (GSH) was measured at 412 nm using the method of Ellman [53], modified by Jollow et al. [54]. The method is based on the development of a yellow color when DTNB (5,5-dithiobtis-2 nitro benzoic acid) is added to the compounds containing sulphydryl groups. Total GSH content was expressed as μg/mg of protein.

Glutathione peroxidase (GPx) activity was determined as described by Flohe and Gunzler [55]. The absorbance at 340 nm was recorded, and the GPx enzyme activity was expressed as nmoles of GSH oxidized/min/mg protein.

##### Determination of Lactate Dehydrogenase Activity

Lactate dehydrogenase (LDH) activities in bone, plasma, and erythrocytes were assayed spectrophotometrically using a commercial reagent kit purchased from Biomaghreb (Ariana, Tunisia: Ref: 20012). The activity of LDH was determined by the variation of OD at 340 nm and was expressed as units/tissue/L or units/L, respectively.

##### Histological Studies

Femurs, intended for histological examination, were taken and immediately demineralized for 72 h in acetic acid (1.7 mol/L) and referred to Talbot et al. [56]. Then they were fixed for 48 h in 10% of formalin solution, embedded in paraffin, serially sectioned at 5 μm, and finally stained with hematoxylin-eosin for light microscopy examination.

##### DNA Fragmentation and Quantification Analysis

Total DNA was extracted from WBC and bone referring to the method of Kanno et al. [57]. After that, DNA fragmentation was assayed by electrophoresis on an agarose gel, as described by Sellins and Cohen [58]. All determinations were performed in triplicate.

### 4.11. Statistical Analysis

The data were analyzed using the statistical package program Stat view 5 Software for Windows (SAS Institute, Berkley, CA, USA). Statistical analysis was performed using one-way Analysis of Variance (ANOVA) followed by Fisher’s Protected Least Significant Difference (PLSD) test as a post hoc test for comparison between groups. All values were expressed as means ± S.D. Statistical tests were 2-tailed, and *p* ≤ 0.05 was considered significant.

## 5. Conclusions

Our in vitro and in vivo studies revealed that the supplementation of FRE improved osteomineral metabolism, bone histo-architecture, and antioxidant defense against free radicals in bone and blood. This is the first report on the protective potentials of this alga against MT hematotoxicity, genotoxicity, and oxidative damage in mammalian bone and blood. In conclusion, the present data might contribute to the rational use of red marine algae as a possible therapy for diseases related to oxidative stress owing to its high nutritional value and putative curative properties.

## Figures and Tables

**Table 1:** Antibacterial power of the tested alga against seven bacteria.

Bacteria	Diameter of Inhibition Zone (mm)
*S. Typhimurium*	6.5 ± 0.25 ^a^
*K. Pneumonia*	7.25 ± 0.2 ^b^
*L. Monocytogenes*	9.5 ± 0.21 ^b^
*Actinomyces* sp.	11 ± 0.25 ^c^
*E. Faecalis*	8.5 ± 0.5 ^b^
*Enterobacteria*	6.5 ± 0.25 ^a^
*M. luteus*	6.5 ± 0.25 ^a^

Results are expressed as the mean of three experiments ± SD. The number of determinations was n = 3; ^a,b,c^ indicate significant differences in the diameter of the inhibition zones of different bacteria (*p* < 0.05).

**Table 2:** Initial and final body weights, absolute and relative femur weights, daily food and water consumption by control and treated rats with MT and MT co-administrated with FRE for 7 days.

Parameters	Treatment Groups			
	Control	MT	MT + Alga	Alga
Initial body weights (g)	254 ± 3.01 ^a^	250 ± 3.07 ^a^	255 ± 3.1 ^a^	260 ± 3.13 ^b^
Final body weights (g)	261 ± 3.05 ^a^	232 ± 2.98 ^c^	239 ± 3.12 ^b^	269 ± 3.01 ^a^
Absolute femur weights (g)	0.72 ± 0.13 ^c^	0.56 ± 0.06 ^b^	0.67 ± 0.18 ^a^	0.67 ± 0.11 ^a^
Relative femur weight (g/100 g BW)	0.27 ± 0.01 ^a^	0.24 ± 0.02 ^a^	0.28 ± 0.03 ^a^	0.25 ± 0.07 ^a^
Food consumption (g/day/rat)	17.15 ± 0.67 ^c^	13.10 ± 1.73 ^a^	16.008 ± 0.53 ^b^	16.07 ± 1.05 ^c^
Drinking water intake (ml/day/rat)	15.95 ± 0.87 ^c^	12.25 ± 0.67 ^a^	13.98 ± 1.03 ^b^	14.85 ± 1.42 ^c^

Results are expressed as the mean of three experiments ± SD. The number of determinations was n = 6. ^a,b,c^ in the same line indicate significant differences in experimental groups (*p* < 0.05).

**Table 3:** Effect of daily administration of MT and alga on hematological parameters in different experimental groups of rats.

Parameters	Treatment Groups			
	Control	MT	MT + Alga	Alga
RBC count (10^6^/mL)	8.16 ± 0.17 ^c^	6.39 ± 0.24 ^a^	7.49 ± 0.09 ^b^	8.15 ± 0.20 ^c^
Hb (g/dL)	13.82 ± 0.32 ^c^	9.58 ± 0.19 ^a^	13.32 ± 0.23 ^b^	13.80 ± 0.35 ^c^
HT (%)	43.23 ± 0.34 ^a^	47.16 ± 0.35 ^c^	44.38 ± 0.33 ^b^	43.22 ± 0.30 ^a^
MCV (mm^3^/RBC)	52.66 ± 0.373 ^a^	52.21 ± 0.33 ^a^	52.40 ± 0.683 ^a^	52.66 ± 0.302 ^a^
MCH (pg/RBC)	16.60 ± 0.33 ^a^	16.37 ± 0.25 ^a^	16.46 ± 0.33 ^a^	16.58 ± 0.379 ^a^
MCHC (g/dL)	31.82 ± 0.408 ^a^	32.33 ± 0.25 ^a^	31.89 ± 0.343 ^a^	31.92 ± 0.443 ^a^
PLT (10^3^/μL)	855.45 ± 3.36 ^d^	761.94 ± 3.34 ^a^	804.46 ± 3.43 ^b^	838.69 ± 3.65 ^c^
WBC count (10^3^/mL)	11.84 ± 0.33 ^a^	18.78 ± 0.56 ^c^	14.28 ± 0.59 ^b^	11.86 ± 0.38 ^a^

Results are expressed as the mean of three experiments ± SD. The number of determinations was n = 6. ^a,b,c,d^ in the same line indicate significant differences between experimental groups (*p* < 0.05).

**Table 4:** Mineral content levels in the bone, blood, and urine of controls and rats treated with MT, MT + alga, and alga after 7 days.

Parameters	Treatment Groups			
	Control	MT	MT + Alga	Alga
Plasma levels (mg/L)				
Calcium (Ca)	60.23 ± 2.18 ^a^	84.67 ± 4.43 ^c^	71.12 ± 2.55 ^b^	65.15 ± 1.18 ^a^
Phosphorus (P)	60.13 ± 0.97 ^a^	63.08 ± 1.70 ^b^	60.45 ± 0.49 ^a^	60.02 ± 0.88 ^a^
Sodium (Na)	138.67 ± 1.53 ^a^	141.67 ± 3.06 ^a^	138.33 ± 1.53 ^a^	139.24 ± 0.51 ^a^
Magnesium (Mg)	0.72 ± 0.04 ^a^	0.85 ± 0.13 ^a^	0.72 ± 0.03 ^a^	0.73 ± 0.02 ^a^
Potassium (K)	150.88 ± 2.30 ^a^	165.94 ± 2.93 ^c^	158.73 ± 1.62 ^b^	161.33 ± 3.06 ^c^
Bone levels (mg/g)				
Calcium (Ca)	143.05 ± 3.07 ^c^	53.3 ± 5.22 ^a^	136.61 ± 3.59 ^b^	144.96 ± 4.74 ^c^
Phosphorus (P)	52.6 ± 2.36 ^c^	24.89 ± 3.89 ^a^	40.96 ± 2.37 ^b^	35 ± 4.41 ^b^
Sodium (Na)	63.17 ± 2.03 ^c^	56.71 ± 1.69 ^a^	58.62 ± 1.09 ^b^	60.02 ± 1.46 ^b^
Magnesium (Mg)	35.69 ± 2.11 ^a^	43.23 ± 0.76 ^b^	42.55 ± 2.02 ^b^	39.92 ± 1.59 ^b^
Potassium (K)	48.11 ± 0.84 ^c^	27 ± 2.36 ^a^	31.83 ± 1.69 ^b^	34.39 ± 2.21 ^b^
Urinary levels (mg/L)				
Calcium (Ca)	82.17 ± 2.00 ^a^	94.86 ± 2.88 ^b^	79.70 ± 1.50 ^a^	80.79 ± 2.50 ^a^
Phosphorus (P)	216.16 ± 4.51 ^a^	604.83 ± 7.03 ^c^	304.27 ± 6.40 ^b^	225.69 ± 4.10 ^a^
Sodium (Na)	84.06 ± 3.68 ^c^	38.05 ± 2.03 ^a^	73.52 ± 3.63 ^b^	81.40 ± 4.24 ^c^
Magnesium (Mg)	25.71 ± 0.50 ^b^	22.65 ± 0.83 ^a^	23.95 ± 0.83 ^a^	25.94 ± 0.79 ^b^
Potassium (K)	239.33 ± 4.04 ^d^	172.67 ± 4.03 ^a^	214.20 ± 3.94 ^b^	225.43 ± 1.25 ^c^

Results are expressed as the mean of three experiments ± SD. The number of determinations was n = 6; ^a,b,c,d^ in the same column indicate significant differences between experimental groups (*p* < 0.05).

**Table 5:** Oxidative stress markers in erythrocytes and bone of adult rat controls and treated with MT, alga or their combination (MT + alga) for 7 days.

Parameters	Treatment Groups			
	Control	MT	MT + alga	Alga
Erythrocyte				
MDA (nmoles MDA/g tissue)	51.12 ± 2.69 ^a^	80.56 ± 2.76 ^c^	66.93 ± 1.91 ^b^	51.07 ± 0.34 ^a^
AOPP (μmoles/mg protein)	1.56 ± 0.26 ^b^	1.86 ± 0.39 ^b^	1.43 ± 0.18 ^a^	1.61 ± 0.06 ^b^
GSH (μg/mg of protein)	50.44 ± 4.79 ^b^	41.27 ± 3.47 ^a^	48.53 ± 3.1 ^b^	51.05 ± 4.26 ^b^
CAT (μmoles H_2_O_2_ degraded/min/mg protein)	3.75 ± 0.49 ^b^	2.25 ± 0.22 ^a^	3.81 ± 0.38 ^b^	3.45 ± 0.55 ^b^
SOD (units/mg protein)	26.88 ± 0.89 ^c^	12.18 ± 0.54 ^a^	16.15 ± 0.73 ^b^	26.83 ± 0.73 ^c^
GPx (nmoles of GSH/min/mg protein)	4.13 ± 0.10 ^c^	2.16 ± 0.03 ^a^	3.15 ± 0.10 ^b^	4.14 ± 0.07 ^c^
Bone				
MDA (nmoles MDA/g tissue)	25.34 ± 1.07 ^a^	42.06 ± 0.86 ^d^	30.08 ± 0.58 ^c^	27.32 ± 0.58 ^b^
AOPP (μmoles/mg protein)	0.32 ± 0.04 ^a^	0.45 ± 0.06 ^b^	0.31 ± 0.14 ^a^	0.43 ± 0.05 ^b^
GSH (mg/g tissue)	67.41 ± 2.81 ^b^	56.024 ± 2.18 ^a^	64.14 ± 2.81 ^b^	66.88 ± 3.02 ^b^
CAT (μmoles H_2_O_2_ degraded/min/mg protein)	14.91 ± 0.49 ^a^	7.73 ± 1.95 ^b^	10.11 ± 0.26 ^a^	14.36 ± 1.26 ^a^
SOD (units/mg protein)	19.57 ± 6.22 ^a^	12.17 ± 5.44 ^b^	17.81 ± 5.27 ^a^	19.18 ± 6.31 ^a^
GPx (nmoles of GSH/min/mg protein)	11.74 ± 1.57 ^c^	6.18 ± 1.21 ^a^	9.1 ± 1.91 ^b^	10 ± 0.70 ^b^

Results are expressed as the mean of three experiments ± SD. The number of determinations was n = 6; ^a,b,c,d^ in the same column indicate significant differences between experimental groups (*p* < 0.05).

## Data Availability

Data is contained within the article.
